# In Vitro Evaluation of Zinc Oxide Tetrapods as a New Material Component for Glaucoma Implants

**DOI:** 10.3390/life12111805

**Published:** 2022-11-07

**Authors:** Svenja Rebecca Sonntag, Stefanie Gniesmer, Anna Gapeeva, Klaus Jakob Offermann, Rainer Adelung, Yogendra Kumar Mishra, Ala Cojocaru, Sören Kaps, Swaantje Grisanti, Salvatore Grisanti, Aysegül Tura

**Affiliations:** 1 Department of Ophthalmology, University of Luebeck, 23538 Luebeck, Germany; 2 Institute for Materials Science, Christian-Albrechts-University of Kiel, 24118 Kiel, Germany; 3 Mads Clausen Institute, NanoSYD, University of Southern Denmark, 6400 Sønderborg, Denmark; 4 Phi-Stone AG, 24143 Kiel, Germany

**Keywords:** glaucoma filtering surgery, nanotechnology, ZnO tetrapods, postoperative encapsulation, antiproliferative substances

## Abstract

**Simple Summary:**

Glaucoma is a disease with an increasing number of sufferers and carries the risk of blindness. The main risk factor is the intraocular pressure. For this reason, eye drops and surgeries are available for treatment. Unfortunately, the tissue surrounding glaucoma stents, in which the aqueous humor is drained through an additional drainage path under the conjunctiva, carries the risk of scarring of such a bleb. This work therefore deals with the investigation of a new implant material, which should prevent this scarring and thus secure the operation success in the long term. For this purpose, implant materials consisting of polydimethylsiloxane (PDMS) and tetrapodal zinc oxide (ZnO-T) nanoparticles were investigated. It has been shown in previous studies that ZnO-T particles have the potential to prevent this scarring.

**Abstract:**

In our previous study we were able to show that zinc oxide (ZnO) tetrapods inhibit wound healing processes. Therefore, the aim of this study was to test the antiproliferative effect of two types of porous polydimethylsiloxane (PDMS)/ tetrapodal zinc oxide (ZnO-T) materials, as well as their usability for glaucoma implants. To find the best implant material, two different porous PDMS/ZnO-T materials were examined. One consisted of 3D interconnected PDMS coarse-pored foams with protruding ZnO-T particles; the other consisted of fine-pored 3D interconnected ZnO-T networks homogeneously coated by a thin PDMS film in the nanometer range. Fibroblast cell viability was investigated for both materials via MTT dye, and some implant material samples were further processed for electron microscopy. Both PDMS/ZnO-T materials showed reduced cell viability in the MTT staining. Furthermore, the electron microscopy revealed barely any fibroblasts growing on the implant materials. At the surface of the fine-pored implant material, however, fibroblasts could not be observed in the etched control samples without ZnO-T. It was found that post-processing of the material to the final stent diameter was highly challenging and that the fabrication method, therefore, had to be adapted. In conclusion, we were able to demonstrate the antiproliferative potential of the two different PDMS/ZnO-T materials. Furthermore, smaller pore size (in the range of tens of micrometers) in the implant material seems to be preferable.

## 1. Introduction

Glaucoma is a chronic disease, which can cause blindness in its later stages. Worldwide, it is the second most frequent cause of bilateral blindness. In 2020, 5.9 million people [1], out of a total number of 76 million glaucoma cases, suffered from bilateral blindness [2]. The main target for improving the visual outcome is the intraocular pressure (IOP) [3,4,5,6,7,8,9]. The Early Manifest Glaucoma Trial in 2002 showed that lowering IOP by 25 percent led to a significantly lower progression rate, compared with that of a control group with non-treatment [10]. First-line therapy consists either of eyedrops or selective laser trabeculoplasty [11], but both therapies are limited in several cases by either reduced adherence or progressive glaucoma. 

Therefore, glaucoma surgery plays an important role in the successful treatment of patients. In addition to the well-known trabeculectomy, the advantages of microshunts—such as the reduction of surgical risks (especially postoperative hypotony) and the reduction of postoperative inflammation, scar formation, or cataract induction—are often discussed [12,13,14].

Newer studies show that the PRESERFLO™ MicroShunt, for example, which drains aqueous humor into the subconjunctival space, lowers IOP, while postoperative management was less intensive than in patients with conventional trabeculectomy [15]. Nevertheless, postoperative success is still limited by fibrous encapsulation [16,17]. Currently, in order to avoid the postoperative increase in IOP, antiproliferative drugs such as mitomycin c (MMC) or 5-fluorouracil (5-FU) are used during operations and postoperatively [18]. Both drugs can cause corneal epithelial toxicity [18,19] or late-onset leak (>3 months after surgery) with bleb ischemia and breakdown of the conjunctiva, followed by postoperative hypotony and its complications [14]. 

For this reason, various clinical studies have explored the relationship of fibrosis and stent material [16,20,21,22]. It has been shown that a rough surface of an implant leads to increased cell adhesion and, thus, to fibrous encapsulation [16]. Furthermore, polypropylenes and vivathanes seem to be associated with stronger inflammation than silicone [21,23]. Newer studies focus on the opportunity of coating the drainage devices with antiproliferative drugs to avoid postoperative fibrosis [24]. These studies show that the right material plays a crucial role in the development of new microshunts.

In terms of new materials, the current focus is often on nanotechnologies [25]. There have been some studies showing the potential success of nanotechnologies in glaucoma surgery [26,27]

In our previous study, we were able to show that ZnO tetrapods (ZnO-T), which are new forms of ZnO nanoparticles [28], inhibit wound-healing processes, such as fibroblast proliferation, migration, transdifferentiation, and cytokine release [29]. 

As a result of the studies mentioned above, including our own previous work, we created two types of porous PDMS/T-ZnO materials and tested their potential as new glaucoma implant materials with antifibrotic properties.

## 2. Materials and Methods

### 2.1. Fabrication Methods of Implant Materials

**Figure 1:**
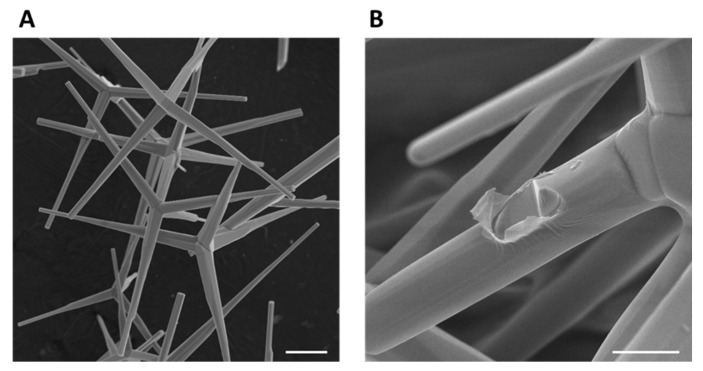
Tetrapodal ZnO particles produced by flame transport synthesis: (**A**) untreated (scale bar = 10 µm); (**B**) coated with a nanometer-thin PDMS film (scale bar = 5 µm).

In this work, tetrapodal-shaped zinc oxide particles (ZnO-T) produced by flame transport synthesis [30] (Figure 1A) at Kiel University were used to create two types of porous PDMS/ZnO-T composite materials.

In the first approach, the direct templating technique, in which porous PDMS foams are prepared by dissolving the sacrificial template (made, e.g., from salt or sugar particles) [31,32] was modified by mixing 20 wt% ZnO-T particles into the template material (salt crystals). Subsequently, the particle mixture was pressed inside a custom-made Teflon mold and liquid PDMS Sylgard 184 (Sigma-Aldrich Chemie GmbH, Taufkirchen, Germany) was poured in, followed by degassing in a vacuum desiccator to facilitate the penetration of PDMS and remove air bubbles. Afterward, the samples were cured for at least 2 h at 85 °C in an atmospheric furnace. Finally, the samples were removed from the mold and placed in an ultrasonic bath at water temperature of approximately 70 °C to dissolve the template material. As a result, 3D interconnected PDMS foams with protruding ZnO-T particles were produced. As reference material, ZnO-T free samples, i.e., pure PDMS foam samples, were prepared in the same way using only template material without ZnO-T particles. The samples exhibited coarse pores in the range of hundreds of micrometers.

**Figure 2:**
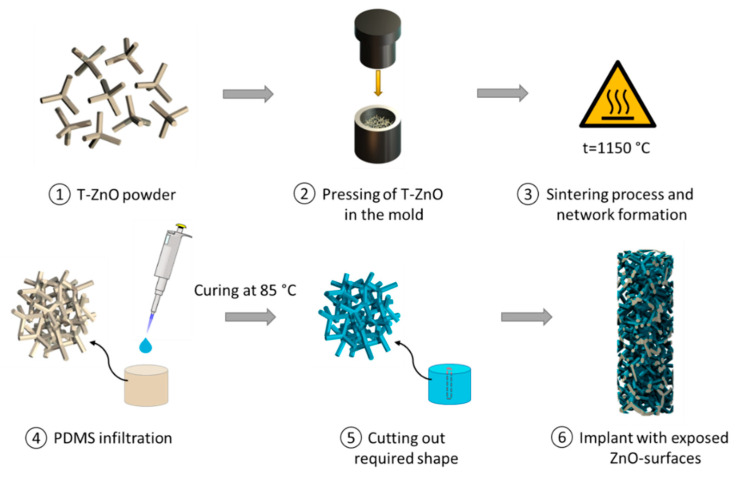
Manufacturing process of PDMS/ZnO-T composite material and resulting implant with exposed ZnO surfaces (shown in grey).

The second approach is demonstrated in Figure 2. To fabricate porous PDMS/ZnO composite material, different amounts of tetrapodal ZnO particles were weighed using a high-precision balance and pressed in a steel mold into cylindrical samples with three different ZnO-T densities of 0.2, 0.3 and 0.4 g/cm³. The pore size of T-ZnO templates was in the range of tens of micrometers [33]. In the next step, the samples were removed from the mold and sintered in a furnace at 1150 °C for 5 h to form interconnected networks. Thereafter, the samples were infiltrated with a PDMS/Hexane (Carl Roth GmbH + Co. KG, Karlsruhe, Germany) solution at a 1:9 weight ratio five times, with intervals of 15 min to allow the solvent to evaporate. Finally, the samples were cured for at least 2 h at 85 °C in an atmospheric furnace. As a result, ZnO networks were homogeneously coated by a thin PDMS film in the nanometer range, as can be seen in Figure 1B. Eventually, the implant of the required dimensions can be cut out (Figure 2, step 5) of the prepared material using laser technology. Cutting creates exposed ZnO surfaces on the surface of the implant, where cell-inhibiting properties are required. For in vitro testing, the samples were cut using a sharp blade, and the surface area with exposed ZnO surfaces was approximately 28 mm². To produce reference samples without ZnO-T, the samples with 0.3 g/cm³ ZnO-T density were etched with 60% acetic acid solution for 1 and 5 days.

For both approaches, the samples had a cylindrical shape with a diameter of 6 mm and the height of 3 mm; they are called implants in the following discussion. 

### 2.2. Establishment of Human Tenon’s Fibroblast (HTF) Cultures

Samples of the human Tenon’s capsule were obtained from patients undergoing cataract surgery at the Tuebingen University Hospital. The cell line was kindly provided by the Tuebingen University Hospital and used for our experiments. The study was in accordance with the tenets of the Declaration of Helsinki for the use of human tissue and informed consent was obtained from the patients after explanation of the nature and possible consequences of the study. The study was approved by the ethics committee of the Tuebingen University Hospital (date of approval: 09.04.2003, project number: 90/2003V). The generation of HTF cultures was performed as described previously [34]. Briefly, the tissue was dissected into 1 mm to 2 mm cubes and maintained in DMEM/F-12 (1:1) medium supplemented with 10% heat-inactivated fetal calf serum (FCS, Invitrogen-Gibco Life Technologies, Karlsruhe, Germany), 2 mM L-glutamine, 100 U/mL penicillin, and 100 µg/mL streptomycin (Biochrom, Berlin, Germany) in a 100 mm Petri dish at 37 °C in a humidified atmosphere with 5% CO_2_. The fibroblasts migrating from these tissues were harvested after approximately 3 weeks by incubation with 0.05% trypsin and 0.02% EDTA (Invitrogen), centrifuged at 300 g for 8 min, and seeded in fresh culture medium in 75 cm^2^ flasks. The cells between the third and seventh passages were used for the experiments.

### 2.3. Co-Incubation of the HTFs with the Implants 

The implants were disinfected by incubating in 70% ethanol three times for 10 min, followed by rinsing in sterile distilled water three times for 5 min and air-drying under a laminar flow hood. Incubation of the HTFs with the implants was performed under two different experimental designs with the above-explained different materials. 

In the first setup, where the fine-pored PDMS/ZnO-T composite material was used, we were interested in determining the viability of both of the cells that were in direct contact with the implants, as well as the cells in the periphery. For this purpose, the implants were centered in the wells of a 24-well plate and the HTFs were seeded at a volume that was sufficient to cover the implant surface (80.000 cells/500 µL/well), taking care not to dislocate the implants. After overnight attachment in a humidified incubator at 37 °C with 5% CO_2_, the implants were immobilized by carefully inserting a silicone ring on top, followed by the addition of 1 mL of fresh medium, and the cells were incubated further for 2 days. The silicone ring and the implants were then removed. The implants were processed for electron microscopy or staining with MTT dye, whereas the viability of the cells that remained in the wells was assessed by MTT assay as described in the following section. 

In the second setup, a silicone ring was initially placed onto the bottom of each well of a 24-well plate and the implants were inserted into the middle of each ring. For this experiment, the coarse-pored PDMS/ZnO-T foams were used.

The HTF cells were seeded onto the implants (32.000 cells/1 mL/well) and incubated for 3 days at 37 °C with 5% CO_2_. As a background control for the MTT treatment, an additional group of implants received only the culture medium without cell seeding. Afterwards, the implants were either processed for electron microscopy or treated with the MTT dye to detect cell growth on the implant surface as described below. 

### 2.4. MTT Assay

The MTT reagent was utilized for both the quantification of metabolically active cells that remained in the periphery of the implants and the assessment of cell growth on the implant surface. For the former purpose, the implants were removed and the cells that remained in the wells were incubated for 3 h with the MTT dye (Sigma-Aldrich, Darmstadt, Germany), which was administered into the culture medium at a final concentration of 0.5 mg/mL. As background control, a separate group of cells were maintained in the culture medium without the MTT dye. Afterwards, the incubation medium was completely removed and the cells were lysed in dimethylsulfoxide for 15 min on a shaker at 600–700 rpm. The lysates were transferred into a 96-well plate in triplicate and the absorbance at 544 nm was measured using microplate readers (FLUOstar Optima, BMG Labtech, Ortenberg, Germany; Molecular Devices, Munich, Germany). The mean absorbance was determined for each group after background substraction.

To demonstrate the extent of cell growth on the explants, the cell-seeded and control implants were incubated for 3 h with the MTT dye, which was administered into the culture medium at a final concentration of 0.5 mg/mL. After the complete removal of the incubation medium, images of the culture wells were acquired using a digital camera (Canon A2500, Krefeld, Germany). The extent of MTT staining on the implants was quantified by measuring the pixel intensity (gray values) of the selected areas using the Image J software (National Institutes of Health, Bethesda, MD, USA, http://imagej.nih.gov/ij/, 1997–2012, accessed on 21 October 2022) [25]. The gray values (0: black, 255: white) were reciprocated to assign higher intensity values to darker stainings. For the normalization of the data from independent experiments that were documented under different ambient illuminations, the intensity values of the control and ZnO-T samples were presented as the fold change relative to the negative control of each experiment. 

### 2.5. Scanning Electron Microscopy (SEM)

The implants were fixed in 4.5% formaldehyde for 30 min and rinsed in distilled water for 10 min. Dehydration was performed by incubating the samples in an ascending series of ethanol (50-60-70-80-90-95%) for 10 min each, followed by three incubations in 100% ethanol for 10 min and immersing in Hexadimethyldisilazane for 3 min [35]. The samples were then air-dried on blotting paper and processed for electron microscopy. Micrographs were obtained using the SEM microscope Zeiss Ultra Plus with the Gemini column (Carl Zeiss Microscopy GmbH, Jena, Germany) at 5 kV acceleration voltage. The PDMS/T-ZnO and pure PDMS samples were sputtered with a conductive layer of gold for 90 s at 30 mA using the sputter coater BAL-TEC SCD 050 (Bal-Tec AG, Pfäffikon, Switzerland).

### 2.6. Statistical Analysis

Data were analyzed with the NCSS statistical software (version 22.0.3, Kaysville, UT, USA). The normality and equal variance assumptions were verified by the Shapiro–Wilk and variance–ratio tests, respectively. Comparison of the mean values of normally distributed continuous variables between two independent groups was performed by using the two-sided *t*-test for unpaired samples with equal variance. In the absence of a normal distribution, the non-parametric Mann–Whitney U test was utilized for the comparison of outcomes among two independent groups. *p* values below 0.05 were considered as significant. Data were presented as the mean ± standard deviation (SD) of n = 3–4 independent experiments.

## 3. Results

### Optimization of the Implant Composition

To determine the optimal constitution of a material that would be unpermissive to cell growth, we initially analyzed the viability of the HTF cells after incubation on the surface of the implants. For this purpose, slices of the fine-pored implants, which occupied approximately 1/6 of the well area of a 24-well plate, were placed into the center of each well, with the cut side (with exposed ZnO surfaces) facing upwards, and the HTF cells were allowed to grow in the culture wells for 3 days. Afterwards, the implants were removed and the extent of the metabolically active cells that remained in the wells was determined by the MTT test. The amount of cell growth on the implant surface and/or interior was assessed by the MTT staining and electron microscopy. Additional sets of experiments were performed by culturing the HTF cells for 3 days in duplicate on the coarse-pored implants, which were stabilized at the bottom of the culture wells using silicone rings. A group of the coarse-pored implants were processed for electron microscopy, whereas the remaining implants were incubated with the MTT dye to macroscopically detect the extent of cell growth on the implant surface.

A total of n = 15 implants with different compositions and ZnO-T concentrations, as well as n = 6 control implants without the ZnO-T, were screened by these in vitro approaches. Both implant materials with the ZnO-nanoparticles showed reduced staining with the MTT dye.

**Figure 3:**
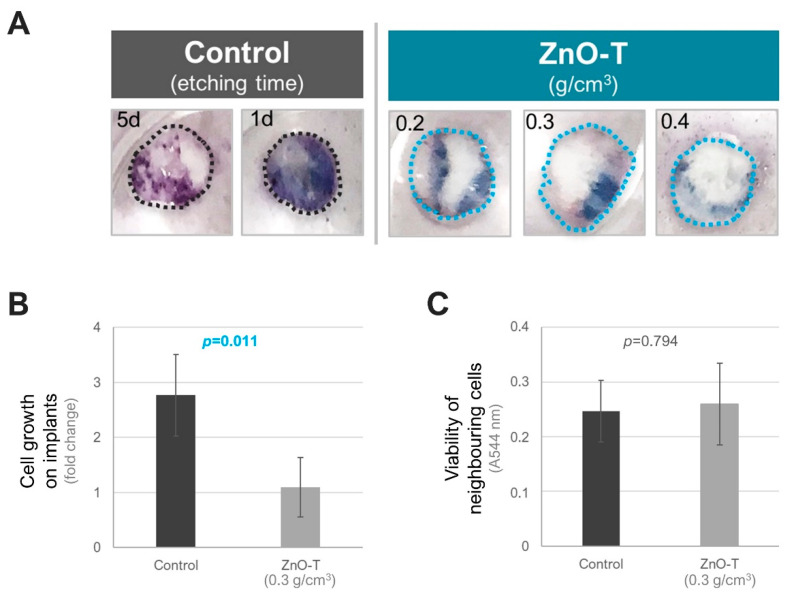
The fine-pored implants with ZnO-Tetrapods (ZnO-T) are unpermissive to the growth of HTF cells on their surface. (**A**) MTT staining of the implants that were used as a substrate for the seeding of the HTF cells. The staining intensity was higher on the control samples that were etched for 1–5 days (d) as opposed to the implants that contained ZnO-T in varying densities (g/cm³). The implants were marked by the dashed lines. (**B**) Quantification of the MTT intensity on the implants with ZnO-T (0.3 g/cm^3^) compared to the controls that were etched for 1 day (mean ± standard deviation (SD) of n = 4 experiments). Data are presented as the fold change relative to the negative control of each experiment. (**C**) Viability of the cells that remained in the periphery of the implants as detected by the MTT assay (mean ± SD of n = 4 experiments). A: absorbance, nm: nanometer. The *p*-values were determined by the two-sided *t*-test with equal variance.

In detail, the implants of the fine-pored material exhibited a lower level of staining with the MTT dye, compared with the etched samples after the incubation with the HTF cells, suggesting the suppression of cell growth on the surfaces with the ZnO-nanoparticles (*p* = 0.011, two-sided *t*-test with equal variance, Figure 3A,B). 

However, no notable difference was observed in the viability of the cells that remained in the periphery of the ZnO-T implants, compared with the controls, suggesting that the ZnO-nanoparticles do not exert toxic effects on the surrounding cells that are not in direct physical contact with these materials (*p* = 0.794, two-sided *t*-test with equal variance, Figure 3C).

**Figure 4:**
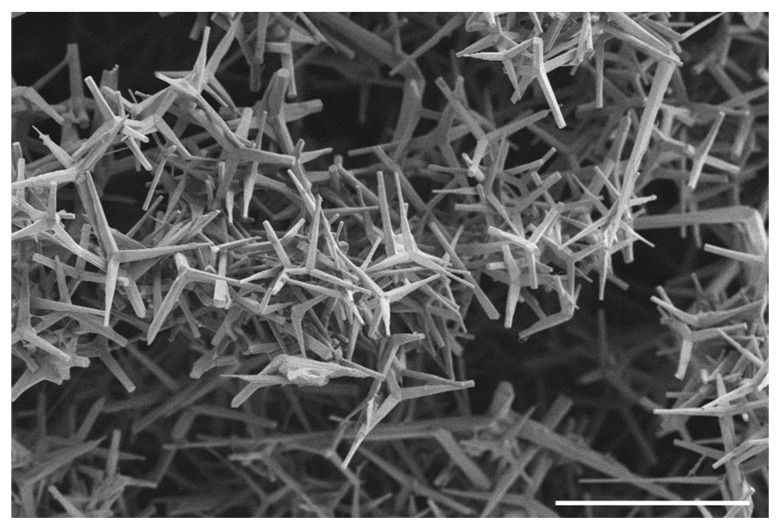
Electron microscopy image of the ZnO-T network coated with PDMS (scale bar = 100 µm). There were no fibroblasts growing on the surface of the fine-pored PDMS/ZnO-T material.

**Figure 5:**
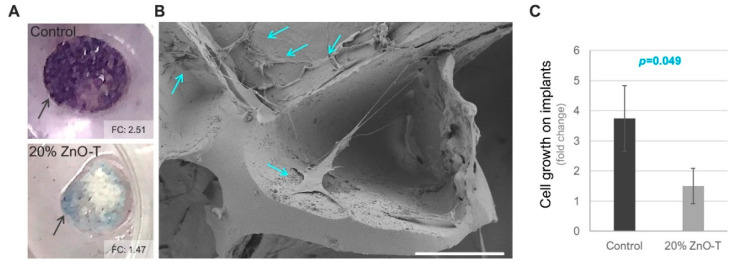
Impaired growth of the HTF cells on the coarse-pored implants with ZnO-T particles. (**A**) MTT staining of the implants that were used as a substrate for the seeding of the HTF cells. A higher level of metabolically active cells could be detected on the implant without the ZnO-nanoparticles (control) compared to the sample with 20% ZnO-T. Arrows indicate the implants that were stabilized at the bottom of the culture wells using silicone rings prior to cell seeding. The staining intensity of the implants was stated as the fold change (FC) relative to the negative control of each experiment. (A) The electron microscopic analysis of the control sample in (**B**) demonstrated the growth of HTF cells (blue arrows) in the cavities. Scale bar = 100 µm. (**C**) Quantification of the MTT staining as an indicator of cell growth on the implants (mean ± standard deviation of n = 3 experiments). The *p*-value was determined by the Mann–Whitney U test.

Electron microscopy images confirmed those results. The images did not show significant growing of fibroblasts on the surface of the fine-pored ZnO-T implants (Figure 4). In contrast to coarse-pored ZnO-T-free samples (Figure 5B), no fibroblasts were found on the surfaces of fine-pored ZnO-T-free samples (data not shown), despite intensive staining in the MTT test (Figure 3A,B). As PDMS is a well-known fouling-release material [36,37], the small pore size and thin walls, as well as the resulting softness of the surface, could lead to a weak attachment of the cells and, therefore, facilitate the detachment of cells during fixation, dehydration, and drying procedures, where samples are subjected to multiple liquid exchanges.

The second experimental setup with the coarse-pored material also revealed reduced staining with the MTT assay on the implants with ZnO-T and here, the electron microcopy confirmed the results (Figure 5). The images demonstrate the adhesion and growth of HTF cells on the implants without the ZnO-T (Figure 5B), which also exhibited an intense MTT-staining (*p* = 0.049, Mann–Whitney-U test, Figure 5A,C).

## 4. Discussion

In our study, we investigated two different materials for later use as implants in glaucoma filtering surgery that may prevent fibroblast cell growth and, thus, postoperative fibrosis.

Even though there are already many studies dealing with the prevention of postoperative scarring of the subconjunctival bleb, no optimal solution has yet been found. Cytotoxic drugs, such as MMC or 5-FU, are quite effective, but have certain risks (e.g., epithelial toxicity). In addition, 5-FU often must be used postoperatively in further operations, which is associated with a renewed operative risk and which can be extremely stressful for a patient.

As we were able to show that ZnO-T can inhibit wound-healing processes [29], we were confident that ZnO-T as a material component of a glaucoma implant would be also useful in avoiding postoperative encapsulation.

Furthermore, we were previously able to demonstrate that ZnO-T nanoparticles will not be cytotoxic for the surrounding cells, even though there are Zn ions measured in the culture medium. Several factors play a role here. First, tetrapodal ZnO structures have less cytotoxic potential than spherical ZnO nanoparticles [38]. Second, ZnO-T exhibit their cytotoxic effect through direct cell contact and only to a small extent through free zinc ions [38]. Third, the structure of ZnO-T is relatively large. Therefore, there is no uptake into the cells [39].

Previous studies were also able to show the antibacterial effect of ZnO nanoparticles [40], for example, on Staphylococcus aureus or Streptococcus agalactiae [41,42], which can also prevent postoperative bleb infection and endophthalmitis. 

The implants’ second material was PDMS, a silicone, which also has less inflammatory potential than polypropylene, for example [21]. Therefore, the combination of both, silicone and ZnO-T might be even more powerful in the prevention of scar formation. 

Our results showed that the implants containing ZnO-T also lead to reduced HTF cell growth in the implants. The number of experiments was limited, but the accordance of our current results with our earlier findings provides further support regarding the growth-suppressing effects of the ZnO-T-containing implants. Nevertheless, we should keep in mind that several studies showed the limitations of the MTT test [43]. For instance, the conversion of the yellow MTT salt into the violet-blue formazan crystals is highly dependent on cellular metabolic activity, which may confound the quantification of viable cells. In other words, a low number of cells with a very high metabolic activity may produce a similar amount of formazan crystals from the MTT salt, compared with a high number of metabolically dormant cells. Therefore, it is not possible to interpret the extent of MTT metabolization as a direct indicator of cell quantity [43]. However, we used a control group and electron microscopy to confirm our results.

Having a look at the electron microscopy images, the different growth behavior of the fibroblasts was obvious. Especially in the comparison of the etched samples, the material with finer pores did not show significant cell growth on the implant’s surface, whereas fibroblasts could be seen in the coarse pores of the foam material. These results indicate that pore size might be relevant for the antiproliferative effect of glaucoma implants.

Therefore, it would be desirable to create glaucoma implants out of PDMS/ZnO-T material with finer pores. However, CO_2_ laser cutting of the material to smaller dimensions resulted in the PDMS decomposition at the cut edges, due to high temperatures of the beam needed to cut through ZnO-T arms. Moreover, a particular challenge is the precise cutting out of a 6–8 mm long cylinder without affecting the final diameter, due to the conicity of the laser beam. Thus, the post-processing of the material to the final stent diameter of 100–200 µm is highly challenging. Therefore, the fabrication method has to be adjusted to allow direct stent production in desired dimensions, which can be achieved, for example, through 3D printing. However, the surface properties need to be re-examined to ensure that the antifibrotic potential is still present. 

Due to the antiproliferative properties shown, the PDMS/ZnO-T composite materials might be also useful for other medical indications, e.g., tracheobronchial airway stents, as postoperative fibrosis is also a known complication [44,45].

In addition to the small number of investigated implants, this study was limited by the in vitro conditions and the lack of different cytokine levels or other immunosuppressive substances [46]. Several previous studies showed the role of aqueous humor cytokine levels for postoperative outcomes, as well as its chemoattractant potential [47,48,49]. However, we were able to show in our previous study that ZnO-T also leads to a reduction in cytokines and can, therefore, minimize the risk of scarring in this respect as well [29].

In addition, due to the connection of the anterior chamber and the subconjunctival bleb [50], additional fibroblasts could possibly play a role in postoperative scarring. For this reason, it would be helpful to know to what extent, for example, fibroblasts of the trabecular meshwork differ from HTFs or how scleral and conjunctival fibroblasts would behave. In vivo studies are indispensable here, to prove the anti-scarring potential of our implant material. Nevertheless, we assume that ZnO-T would show an antiproliferative effect for the other fibroblasts, as ZnO-T acts against proliferating cells and, apart from this, an earlier study already showed that the main target for postoperative encapsulation of the bleb are HTFs [51]. 

Furthermore, while developing a new implant material, we should keep in mind that glaucoma stents, such as the Ex-PRESS shunt [52] or in particular the CyPass Micro Stent [53,54], showed a reduction of corneal endothelial cells. Therefore, every new glaucoma implant should be validated for its biocompatibility, especially as our implant material has an antiproliferative effect. Nevertheless, ZnO nanoparticles have been described as antiproliferative for rapidly growing cells and not for cells without high proliferating potential. As the corneal endothelial cells cannot divide and proliferate [53], we think that our implant material does not have a particularly high risk for corneal endothelial cell loss.

Despite the efficiency of ZnO-T, it must be considered that ZnO-T particles may also degrade over time and, therefore, lose their antiproliferative potential. The degradation is influenced, among other things, by the pH value, the particle size, and the media composition. Avramescu et al. classified ZnO as biopersistent at neutral pH [55]. As the pH of the aqueous humor is approximately 7 [56], ZnO should be only slightly soluble. Furthermore, the ZnO-T particles used in this study are larger than conventional, commercially available ZnO nanoparticles [39], and as solubility decreases with increasing size [55], degradation might be slowed down. Phosphate ions, however, which are part of several eye drops, can lead to very fast degradation of ZnO, even at neutral pH [57], and should be avoided when using implants containing ZnO. 

Considering all of these aspects, we are quite confident that our implant material with ZnO-T will have an antiproliferative effect over a longer term, or at least in the first postoperative period where the risk of scarring is particularly high. Further studies are necessary to prove the time period of the antiproliferative efficacy.

In summary, in line with our previous study using ZnO-T in solution, we confirmed the antiproliferative effect with the composite material (PDMS/ZnO-T) intended to be used for a new glaucoma implant. Its usability and intended effect need to be shown in our next step, based on preclinical in vivo experiments.

## Data Availability

Data underlying the results presented in this paper are not publicly available at this time but may be obtained from the authors upon reasonable request.

## Patents

Invention notification was made. The patent process is currently running.
